# Herpes Zoster Vaccine Uptake and Active Campaign Impact, a Multicenter Retrospective Study in Italy

**DOI:** 10.3390/vaccines12010051

**Published:** 2024-01-03

**Authors:** Andrea Ceccarelli, Federica Tamarri, Raffaella Angelini, Elizabeth Bakken, Ilaria Concari, Elsa Giannoccaro, Giada Domeniconi, Michela Morri, Chiara Reali, Francesca Righi, Silvia Serra, Gianmaria Semprini, Giulia Silvestrini, Valentina Turri, Davide Gori, Marco Montalti

**Affiliations:** 1 Operative Unit of Hygiene and Public Health-Forlì and Cesena, Department of Public Health, Romagna Local Health Authority, 47522 Cesena, Italygiada.domeniconi@auslromagna.it (G.D.);; 2 Unit of Hygiene and Medical Statistics, Department of Biomedical and Neuromotor Sciences, University of Bologna, 40126 Bologna, Italy; 3 Operative Unit of Hygiene and Public Health-Ravenna, Department of Public Health, Romagna Local Health Authority, 48121 Ravenna, Italy; 4 Operative Unit of Hygiene and Public Health-Rimini, Department of Public Health, Romagna Local Health Authority, 47924 Rimini, Italy

**Keywords:** herpes zoster, shingles, active campaign, catch-up campaign, vaccine hesitancy, vaccine uptake

## Abstract

The Herpes Zoster (HZ) vaccination has proven both safe and effective in alleviating conditions related to HZ, leading to significant cost savings in national healthcare and social systems. In Italy, it is recommended and provided free of charge to individuals aged 65 and older. To achieve broad vaccination coverage, alongside ordinary immunization campaigns, active and catch-up campaigns were implemented. This retrospective observational study aimed to observe the vaccination coverage achieved in the Romagna Local Health Authority (LHA) during the 2023 active campaign, with a secondary goal of assessing the impact of the 2022 catch-up campaign and the 2023 active campaign compared to ordinary campaigns. As of 3 July 2023, an overall vaccine uptake of 13.5% was achieved among individuals born in 1958, with variations among the four LHA centers ranging from 10.2% to 17.7%. Catch-up and active campaigns together contributed to nearly half of the achieved coverage in Center No. 1 and a quarter in Center No. 2. Notably, individuals born in 1957, not included in the Center No. 2 catch-up campaign, reached significantly lower vaccination coverage compared to other cohorts and centers. Analyzing the use of text messages for active campaigns, it was observed that cohort groups did not show substantial differences in text-message utilization for warnings. However, having relatives who had experienced HZ-related symptoms significantly reduced the reliance on text messages as warnings. These results highlighted how catch-up and active campaigns effectively increased vaccine coverage. Nevertheless, differences in uptake among different centers within the same LHA and the limited contribution of other information sources compared to text messages suggest the necessity of designing campaigns involving all available channels and stakeholders to maximize vaccine uptake.

## 1. Introduction

Vaccination holds the potential to significantly mitigate the occurrence of herpes zoster (HZ)-related conditions and the subsequent development of postherpetic neuralgia (PHN), leading to substantial cost savings within national healthcare and social systems [1]. However, the global landscape of HZ vaccination remains characterized by a lack of uniformity and diversity [2,3,4]. This disparity likely stems from the intricate interplay of various socio-environmental factors rather than being solely attainable through individual endeavors [5].

Vaccine hesitancy (VH) is a major constraint in achieving vaccine targets. It is internationally known that there are multiple psychological and socio-demographic factors that can increase VH, such as lack of confidence, uncomfortableness, calculation, and complacency as barriers to influenza vaccine uptake in at-risk groups [6]. Other factors investigated by the European Center for Disease Prevention and Control included “doubts about vaccine safety” and “information deficit” as two of the main determinants of VH [7].

In addition, various patient-related factors that contribute to HZ-specific VH have been identified, including age, gender, education level, economic status, health conditions, access to accurate information, perceived obstacles, belief in disease control without vaccination, and prior history of shingles [8,9]. Furthermore, a sense of societal responsibility and concern for the welfare of others has emerged as a notable determinant of overall vaccine acceptance among the populace [10]. Consequently, a strategic focus on the societal benefits of achieving robust vaccination coverage within public health messaging could potentially address the issue of VH [11,12]. The pivotal role of General Practitioners (GPs) in promoting vaccination is evident, as their counsel has demonstrated a positive influence on individuals’ inclination to receive vaccines. Similarly, personal familiarity with a family member, friend, or acquaintance who has suffered from HZ appears to bolster vaccine acceptance, likely due to an increased awareness of the disease and its consequences [13,14].

The systematic analysis of the underlying determinants of VH is one of the priorities of public health systems and prevention departments, and the World Health Organization Strategic Advisory Group of Experts has emphasized the importance of implementing impactful public health interventions that consider local geographical nuances and cater to specific target populations [15].

Fostering collaborative engagements among stakeholders is crucial. This might involve deeper collaboration with GPs, the implementation of joint educational initiatives, and the establishment of intervention protocols such as ‘catch-up’ and active campaigns. The orchestration of vaccination programs and campaigns should be spearheaded by public health medical services [16]. It is noteworthy that Italy’s Ministry of Health presently does not monitor the uptake rates of HZ vaccines.

In the Italian context, starting in 2017, the administration of the HZ vaccine has been consistently expanded annually to include individuals aged 65 and above. Moreover, individuals have the choice to privately finance the vaccine in subsequent years [17]. Additionally, individuals aged 18 and above who are exposed to heightened risk factors are also eligible for HZ vaccination at no cost [18]. Local Health Authorities (LHAs) in Italy are empowered to initiate active and catch-up vaccination campaigns targeting individuals who have not adhered to the national immunization schedules [19]. A concrete example of this can be seen in the Calabria Region, where eligible 65- and 70-year-olds were offered a combination of HZ and pneumococcal vaccinations [20].

Aligned with the national vaccination program, the Romagna Local Health Authority (LHA) conducts an annual HZ vaccination campaign specifically targeting individuals who are turning 65 in the respective year and have not previously received the vaccine. During 2022, a catch-up campaign, which concentrated on cohorts born in 1955, 1956, and 1957, was carried out due to missed calls during the COVID-19 pandemic, achieving in the Romagna LHA an adherence rate of 11.4–12.4% [21].

The primary objective of this study was to assess the level of compliance with the 2023 HZ vaccination campaign within the Romagna LHA. A secondary aim was to assess the impact of the 2022 catch-up campaign in relation to the activities of the regular campaign.

## 2. Materials and Methods

This observational study retrospectively examined the entirety of the Public Health Department (PHD) across the four divisions within an Italian LHA. The LHA covers a geographical expanse of around 5000 square kilometers and a population of 1,124,896 individuals.

### 2.1. Study Population

In 2023, the active vaccine campaign specifically centered around individuals born in 1958 who had not previously received the herpes zoster vaccine. Meanwhile, the 2022 catch-up campaign focused on individuals born in 1955, 1956, and 1957 due to a response to missed chances for targeted engagement by the LHA during the COVID-19 pandemic.

### 2.2. Study Setting

The HZ active vaccination campaign slightly differed in terms of timelines and strategies between the four participating centers:

- Center No. 1: On 8 March 2023, 5 days prior to the campaign launch, text messages were sent to the campaign cohort. This cohort comprised 2719 individuals born in 1958. Individuals without a valid or known mobile number (*n* = 153) were informed with a phone call. Additionally, At the start of the month, PHD staff sent informative emails to GPs, and a promotional campaign through press and online advertising was executed roughly 2 weeks before the campaign’s commencement. The campaign itself lasted two weeks, from 13 March to 31 March.
- Center No. 2: On 8 March 2023, 5 days prior to the vaccination campaign, text messages were sent to the campaign cohort (*n* = 5117 born in 1958). Individuals without a valid or known mobile number (*n* = 355) were informed with a landline phone call. At the start of the month, PHD staff sent informative emails to GPs, and a promotional campaign through press and online advertising was executed prior to the campaign commencement. Dedicated vaccination sessions were carried out from 13 March to 14 April.
- Center No. 3: On 8 March 2023 (approximately 14 days before the start of the vaccination campaign), text messages were sent to the cohort involved in the campaign (*n* = 4520 born in 1958). The individuals without a valid or known mobile number (*n* = 939) were informed with a landline phone call. At the start of the month, PHD staff sent informative emails to GPs, and a promotional campaign through press and online advertising was executed prior to the campaign commencement. Dedicated vaccination sessions were organized from 16 March to 24 March.
- Center No. 4: On 8 March 2023, approximately 5 days prior to the campaign launch, text messages were sent to the campaign cohort. This cohort comprised 2402 individuals born in 1958. Individuals without a valid or known mobile number (*n* = 192) were informed with a landline phone call. Additionally, at the start of the month, PHD staff sent informative emails to GPs, and a promotional campaign through press and online advertising was executed roughly 2 weeks before the campaign’s commencement. The campaign itself lasted two weeks, from 13 March to 31 March.

The text message, the same for all four LHA centers, was: ‘Ausl Romagna: March 2023 vaccination campaign against Herpes Zoster for those born in 1958. Book now through Cup and CupTel channels” (channels to use for independently scheduling one’s vaccination).

In addition to booking appointments during the dedicated sessions organized throughout the active campaign, every user could continue to schedule appointments throughout the year within sessions organized as part of the PHD’s routine immunizations for the general population.

In all centers, a prerequisite step before administering vaccinations was conducting an interview with a medical doctor or other PHD personnel. This interview aimed to gather participants’ vaccination and medical histories. In all centers, vaccinations for Herpes Zoster were made available, including both the recombinant technology vaccine and the live attenuated vaccine. Different operational approaches (strategies and timelines) of the 2022 catch-up campaign at Centers No. 1 and No. 2 were discussed in a study conducted by the research team in the previous year [21].

### 2.3. Data Extraction

Data pertaining to the 2023 active vaccination campaign were anonymously extracted from participants’ vaccination history records by four independent reviewers (A.C., G.D., S.S., and M.M.) in June 2023. Any disagreements in the extracted data were resolved through discussion with an impartial arbiter (F.R.). The variables extracted from each record included: “Type of HZ vaccine”, “Gender”, “Age”, “Neurological disorders”, “Primary immunodeficiency”, “Allergies”, “Vaccine allergies”, “Iatrogenic immunosuppression”, “Personal history of HZ”, “Family history of HZ”, and “Source of information used to learn about the active vaccination campaign”. Personal and familial histories of HZ were also considered, as they were deemed potential factors influencing vaccine acceptance. Data were collected and managed using Microsoft Excel (Microsoft Corporation).

Aggregate data concerning the uptake achieved during the 2022 catch-up campaign were obtained from a previous research effort conducted by the research team [21]. Additionally, aggregated data (by cohort and gender) concerning the overall coverage rates achieved for each cohort across all four centers in 2022 and the early months of 2023 were extracted from the vaccination registry of the Romagna LHA.

### 2.4. Statistical Analysis

Variables were characterized using both absolute frequencies and percentages. The factors influencing the utilization of a specific information source, namely text messages, to acquire information about the primary form of information, were evaluated through a multivariate analysis. The results of the multivariate analyses are displayed as odds ratios (ORs) accompanied by their standard errors (SE) and a 95% confidence interval (CI). A backward stepwise analysis was carried out to identify the variables to be included in the ultimate multiple logistic regression model, guided by the principles of parsimony and biological plausibility. The threshold for statistical significance was set at *p* < 0.05. All statistical analyses were executed using Stata Statistical Software 15, developed by StataCorp, College Station, TX, USA.

## 3. Results

### 3.1. Main Sample Features

Table 1 presents a comprehensive overview of the study sample’s main characteristics, consisting of a total of 1039 participants across four different centers: No. 1 (*n* = 282), No. 2 (*n* = 419), No. 3 (*n* = 188), and No. 4 (*n* = 150). The participants were categorized by various attributes, including vaccine type, gender, age, pathological anamnesis, history of herpes zoster, and sources of vaccination campaign information. Notably, the vaccine distribution indicated a predominance of live attenuated virus vaccines across all centers, with the highest administration rate seen in Center No. 4 (*n* = 141; 94%). The gender distribution showed slight variations, with male participants comprising 43% in Center No. 3 and 51% in Center No. 4. In terms of age, most participants fell within the targeted cohort (98%) except for a small minority in each center.

When exploring medical histories, allergies were the most common pathological anamnesis variable (ranging from 9% in Center No. 3 to 18% in Center No. 2). Regarding the history of HZ, a significant portion of participants reported having relatives with the infection (ranging from 31% to 45%) and personal experiences of the infection (ranging from 15% to 20%).

The vaccination campaign’s primary sources of information were text messages, accounting for the majority of responses (ranging from 83% to 93%), followed by landline phone calls, family/friends, and general practitioners.

### 3.2. Herpes Zoster Vaccine Uptake and Active Campaign Impact

Among the cohort born in 1958, as shown in Table 2, a cumulative HZ vaccination uptake of 13.5% was achieved in the Romagna LHA, with 1993 individuals vaccinated. The overall uptake represents the ongoing vaccination coverage up to 3 July 2023. Table 2 provides a comprehensive view of vaccination trends among the various centers, highlighting the uptake differences between the active and ordinary vaccination campaigns.

Regarding vaccinations administered during the active campaigns, it is evident that these have had varying impacts on the overall vaccinations across different centers: 10.4% out of 17.7% at Center No. 1, 8.2% out of 13.4% at Center No. 2, 4.2% out of 10.2% at Center No. 3, and 6.2% out of 15.5% at Center No. 4. A subgroup analysis based on gender is also shown, although notable differences are not found.

A comprehensive assessment of the impact of the active and catch-up campaign on the ordinary campaign is presented in Table 3, where data from the catch-up campaigns of Center No. 1 and Center No. 2 involving cohorts born in 1955, 1956, and 1957 are compared with the overall vaccine coverages achieved within these cohorts throughout the year.

It is notable that the percentages of vaccinations administered during the extraordinary campaign are higher in Center No. 1 compared to Center No. 2 for the cohorts born in 1955 and 1956, despite similar overall coverage rates for these two centers. For the cohort born in 1955, Center No. 1 recorded an 11.5% acceptance rate in catch-up vaccinations and 13.2% in ordinary vaccinations, contributing to an overall uptake of 24.7%. In Center No. 2, the corresponding figures were 6.4% for catch-up vaccinations and 17.6% for ordinary vaccinations, resulting in an overall uptake of 24.0%. For the cohort born in 1956, Center No. 1 reported a 10.6% participation rate in catch-up vaccinations and 12.5% in ordinary vaccinations, resulting in an overall uptake of 23.1%. In Center No. 2, 5.5% of individuals engaged in catch-up vaccinations and 16.4% in ordinary vaccinations, leading to an overall uptake of 22.0%.

However, concerning the overall coverage achieved within the cohort born in 1957, while Center No. 1 reached an 11.0% involvement in catch-up vaccinations and 11.5% in ordinary vaccinations, resulting in an overall uptake of 22.5%, in Center No. 2, only 0.4% of individuals were involved in catch-up vaccinations, while 14.2% were involved in ordinary vaccinations, resulting in a more modest overall uptake of 14.7%.

### 3.3. Multiple Regression Analysis

Table 4 provides insights into the factors influencing the utilization of text messages as a warning mechanism for the vaccination campaign, as determined by a regression model. The odds ratios (OR), standard errors (SE), *p*-values, and 95% confidence intervals (C.I.) are listed for each center, history of HZ, and cohort group. In terms of the centers, Center No. 1 serves as the reference point, and it was found that Center No. 2 (OR: 0.66, SE: 0.11, *p* = 0.017, 95% C.I.: 0.47–0.93) and Center No. 4 (OR: 0.44, SE: 0.12, *p* = 0.003, 95% C.I.: 0.25–0.76) displayed a significant decrease in the likelihood of using text messages for campaign warnings compared to Center No. 1. When considering the history of herpes zoster, while personal experiences did not show any significant association with text-message utilization, having relatives who had experienced HZ showed a significant decrease in the likelihood (OR: 0.67, SE: 0.10, *p* = 0.005, 95% C.I.: 0.50–0.89). Furthermore, the cohort groups, represented by ages 55 to 58, did not demonstrate substantial differences in text-message utilization for vaccination warnings.

## 4. Discussion

This study identified the impact of both active and catch-up campaigns on routine immunizations, with text messages serving as the primary informational conduit for the HZ catch-up and active vaccination campaign administered within an LHA. The overall vaccination coverage within the annual targeted cohort of 1958 individuals was observed to be 13.5% as of July 2023. Nonetheless, higher uptake rates were noted in cohorts from previous years, albeit with significant disparities between cohorts and different centers within the LHA, ranging from 14.7% to 24.7%.

Currently, there is a lack of substantial evidence regarding the vaccination coverage rate for Herpes Zoster at both the Italian and international levels. The Italian Ministry of Health does not currently provide vaccination coverage rates for Herpes Zoster but merely indicates an optimal coverage of 50% [22]. Recent international studies have reported varied uptake rates, ranging from approximately 8% in Saudi Arabia to 46.9% in Australia, with a similar uptake observed in Greece (12%) [23,24,25].

Rates achieved during the 2022 and 2023 catch-up and active campaigns allowed us to observe that approximately 50% of all vaccinations conducted in the four centers of the LHA were carried out during these campaigns. This impact became even more evident when considering the case of the 1957 cohort in Center No. 2. In this instance, due to organizational reasons, the cohort had not yet received either the catch-up campaign in 2022 or the active campaign in 2023, resulting in an overall uptake rate of only 14.7%, the lowest among the two centers where the campaigns of the last two years were analyzed. This finding is consistent with the existing scientific literature, which indicates that active and catch-up campaigns are highly efficient approaches to accelerate population protection against vaccine-preventable diseases [26,27], and it aligns with other studies that have measured the impact of extraordinary campaigns on routine campaigns, albeit with the administration of different vaccines [28,29].

The primary source of information contributing to higher vaccination coverage was the receipt of text messages, which served as the main reason for individuals to participate in the active vaccination campaign. This approach corroborates previous findings in the literature that have emphasized the effectiveness of text messages in promoting vaccine adherence [29,30,31]. This observation prompts consideration of potential extensions of this practice to other vaccination programs, particularly among vulnerable individuals with multiple comorbidities [32,33].

It is worth noting that all other sources of information, including advice from GPs, recommendations from family and friends, online resources, news outlets, landline phone calls, and others, demonstrated minimal impact on vaccine acceptance. This revelation encourages the exploration of alternative interventions, such as educational initiatives involving GPs. A substantial body of literature already underscores the positive effect of educational interventions conducted by GPs on vaccine uptake [34,35]. A study conducted in France [36] highlights the constructive influence of consultations with general practitioners on adherence to national vaccination campaigns, particularly in the context of competing vaccination programs, such as those related to COVID-19.

Moreover, it is documented in the literature that information sources like the Internet and news outlets play pivotal roles in vaccination campaigns [37]. This topic necessitates consideration of ways to enhance these information channels for future catch-up campaigns, as a synergistic approach involving all available channels appears to be the most effective strategy [38].

Additionally, we observed in our study that having a relative who had experienced HZ symptoms emerged as a significant motivator for HZ vaccination. This discovery aligns with expectations, given the potential severity of HZ symptoms, and is consistent with existing literature indicating that having a relative with HZ constitutes a driver, particularly among older age groups [39,40]. Furthermore, in two centers, the utilization of text messages as a means of communication was more likely to be the most frequently used source of information compared to other centers.

Variations in vaccine uptake were also observed among different centers. Indeed, as of July 2023, the overall uptake rate for individuals born in 1958 was 13.5%, with fluctuations ranging from 10.2% to 17.7% among the various centers. This outcome is not particularly surprising, given both the geographical expanse of the LHA and the substantial number of individuals under its care. It is well-established that vaccine hesitancy is a phenomenon strongly influenced by local contexts [41,42], and one of the centers, in particular, has historically exhibited greater hesitancy toward vaccinations [43,44]. Nonetheless, this result underscores the need to devise strategies aimed at standardizing uptake rates across the area, potentially through the implementation of specific initiatives in areas with lower uptake.

Furthermore, it is intriguing to note that, in comparison to the vaccine coverage achieved by the cohort born in 1958 as of July 2023, vaccination coverage for HZ tends to increase progressively among cohorts born in previous years. This trend necessitates ongoing monitoring to determine whether it signifies a genuine rise in vaccination adherence among these cohorts or, more likely, reflects the additional time available for the 1955-born cohort to adhere to the vaccination campaign.

While the findings of this study contribute valuable insights into the specific context and population under investigation, several limitations must be acknowledged in relation to external generalizability. The sample, primarily drawn from a specific geographic region and demographic profile, may limit the broader applicability of the results to more diverse populations. Additionally, even if the study employed a four-site design, the four centers employed slightly different methods and timelines in the management of the active and catch-up campaigns. These limitations highlight the need for caution when extending the findings beyond the current study’s scope, emphasizing the importance of future research to explore the generalizability of these results in diverse populations and settings. Furthermore, the pathological anamnesis and history of HZ infection were reliant on self-reporting and were not cross-referenced with previous medical records, potentially introducing reporting bias. Additionally, the personnel responsible for collecting medical histories varied among the four centers. This variability led to instances where patient records were incomplete for certain study variables, as exemplified by the absence of remote pathological history documentation in Center No. 4. However, it is noteworthy that the absence of these data did not significantly impact the core findings of the study. Moreover, to promote consistency in database creation, only four reviewers were involved in the review of vaccination history records. Lastly, it is important to acknowledge that all vaccination uptake rates for the different cohorts are reflective of data updated as of July 2023.

## 5. Conclusions

In conclusion, our study underscores the crucial contribution of active and catch-up vaccination campaigns to achieving vaccination rate targets, albeit with variations observed among distinct cohorts and healthcare centers. Our findings underscored the potential of text-based interventions in the realm of public health initiatives at the local level. Additionally, the disparities in local vaccine uptake highlight the necessity for customized strategies, and the rising vaccine coverage among older cohorts underscores the significance of timely vaccination efforts. This study suggests directing attention in future research towards those who do not come forward for vaccination and could provide valuable insights for those tasked with devising active or catch-up vaccination activities across various tiers of community healthcare.

## Figures and Tables

**Table 1:** Main Characteristics of the study sample.

		Center				Overall (*n* = 1039)
		No. 1 (*n* = 282)	No. 2 (*n* = 419)	No. 3 (*n* = 188)	No. 4 (*n* = 150)	
**Vaccine**	Live attenuated virus	251 (89)	367 (88)	173 (92)	141 (94)	932 (90)
	Recombinant vaccine	31 (11)	52 (12)	15 (8)	9 (6)	107 (10)
**Gender**	Male	121 (43)	190 (45)	95 (51)	70 (47)	476 (46)
	Female	161 (57)	229 (55)	93 (49)	80 (53)	563 (54)
**Age**	65	275 (98)	403 (96)	185 (98)	150 (100)	1013 (98)
	Other	7 (2)	16 (4)	3 (2)	0 (0)	26 (2)
**Pathological Anamnesis**	Neurological disorders	11 (4)	9 (2)	1 (1)	n.a.	21 (2)
	Primary immunodeficiency	12 (4)	13 (3)	2 (1)	n.a.	27 (3)
	Allergies	31 (11)	76 (18)	16 (9)	24 (16)	147 (14)
	Vaccine allergies	0 (0)	1 (0)	0 (0)	n.a.	1 (0)
	Iatrogenic immunosuppression	15 (5)	14 (3)	1 (1)	n.a.	30 (3)
**History of Herpes Zoster**	Personal	41 (15)	64 (16)	14 (8)	30 (20)	149 (15)
	Relatives	88 (31)	162 (41)	82 (45)	65 (43)	397 (40)
**Vaccination Campaign—source of information**	Text message	234 (84)	363 (93)	169 (90)	125 (83)	891 (88)
	General practitioner	7 (3)	5 (1)	1 (1)	3 (2)	16 (2)
	Family/friends	17 (6)	10 (3)	5 (3)	5 (3)	37 (4)
	Web	2 (1)	1 (0)	0 (0)	0 (0)	3 (0)
	News	1 (0)	2 (1)	4 (2)	1 (1)	8 (1)
	Phone call	11 (4)	2 (1)	5 (3)	15 (10)	33 (3)
	Other	7 (3)	4 (1)	4 (2)	1 (1)	16 (2)

**Table 2:** Active and ordinary Herpes Zoster vaccination campaign uptake among 1958 cohort.

Cohort		2023												
		No. 1 (*n* = 2719)			No. 2 (*n* = 5117)			No. 3 (*n* = 4520)			No. 4 (*n* = 2402)			Overall (*n* = 14.791)
		Active	Ordinary	Overall Uptake *	Active	Ordinary	Overall Uptake *	Active	Ordinary	Overall Uptake *	Active	Ordinary	Overall Uptake *	
1958	M	121 (4.5)	79 (2.9)	480 (17.7)	190 (3.7)	182 (3.6)	685 (13.4)	95 (2.1)	112 (2.5)	459 (10.2)	70 (2.9)	103 (4.3)	372 (15.5)	1993 (13.5)
	F	161 (5.9)	119 (4.4)		229 (4.5)	84 (1.6)		93 (2.1)	159 (3.5)		80 (3.3)	119 (5.0)		

* Cohort vaccination coverage until 3 July 2023.

**Table 3:** Catch-up and ordinary Herpes Zoster vaccination campaign uptake.

Cohort	2022–2023					
	Catch-Up	Ordinary	Overall Uptake *	Catch-Up	Ordinary	Overall Uptake *
	Center No. 1 (*n* = 2410)			Center No. 2 (*n* = 4740)		
1955	278 (11.5)	318 (13.2)	596 (24.7)	303 (6.4)	833 (17.6)	1136 (24.0)
	Center No. 1 (*n* = 2631)			Center No. 2 (*n* = 5067)		
1956	280 (10.6)	329 (12.5)	609 (23.1)	278 (5.5)	832 (16.4)	1110 (22.0)
	Center No. 1 (*n* = 2709)			Center No. 2 (*n* = 5504)		
1957	299 (11.0)	311 (11.5)	610 (22.5)	24 (0.4)	784 (14.2)	808 (14.7)

* Cohort vaccination coverage until 3 July 2023.

**Table 4:** Variables associated with the source of information used in a multiple regression analysis (Statistically significant *p*-Value are indicated in bold).

Use of Text Messages as a Warning for the Vaccination Campaign		OR	SE	*p*-Value	95% C.I.
Center	Center No. 1	1			
	Center No. 2	0.66	0.11	0.017	0.47–0.93
	Center No. 3	0.73	0.22	0.30	0.41–1.32
	Center No. 4	0.44	0.12	0.003	0.25–0.76
History of Herpes Zoster	Personal	0.79	0.15	0.213	0.55–1.14
	Relatives	0.67	0.10	0.005	0.50–0.89
Cohort	55	1			
	56	0.89	0.21	0.617	0.57–1.40
	57	0.70	0.20	0.220	0.41–1.23
	58	0.83	0.18	0.524	0.58–1.32

## Data Availability

The data presented in this study are available on request from the corresponding author.
